# Supplementary material for: Drug-Utilization, Healthcare Facilities Accesses and Costs of the First Generation of JAK Inhibitors in Rheumatoid Arthritis
Source: Pharmaceuticals (Basel). 2023 Mar 21;16(3):465. doi: 10.3390/ph16030465 (PMC10058541; doi:10.3390/ph16030465)
Supplement: Supplementary file 1 [file pharmaceuticals-16-00465-s001.zip › pharmaceuticals-2258572-supplementary.pdf]

Drug-Utilization, Healthcare Facilities Accesses and Costs of the First Generation of JAK Inhibitors in Rheumatoid Arthritis

Irma Convertino [1], Valentina Lorenzoni [2], Rosa Gini [3], Giuseppe Turchetti [2], Elisabetta Fini [4], Sabrina Giometto [5], Claudia Bartolini [3], Olga Paoletti [3], Sara Ferraro [1], Emiliano Cappello [1], Giulia Valdiserra [1], Marco Bonaso [1], Corrado Blandizzi [1,6], Marco Tuccori [1,6,*] and Ersilia Lucenteforte [5]

[1] Unit of Pharmacology and Pharmacovigilance, Department of Clinical and Experimental Medicine, University of Pisa, 56126 Pisa, Italy; convertino.irma@gmail.com (I.C.); saraferraro.a@gmail.com (S.F.); emilianocappello@gmail.com (E.C.); giuliavaldiserra@gmail.com (G.V.); bonasomarco@gmail.com (M.B.); c.blandizzi@gmail.com (C.B.)

[2] Institute of Management, Scuola Superiore Sant'Anna, 56100 Pisa, Italy; vlorenzoni9@gmail.com (V.L.); giuseppe.turchetti@santannapisa.it (G.T.)

[3] Tuscan Regional Healthcare Agency, 50100 Florence, Italy; rosa.gini@ars.toscana.it (R.G.); claudia.bartolini@ars.toscana.it (C.B.); olga.paoletti@ars.toscana.it (O.P.)

[4] Medical Specialization School of Pharmacology, University of Pisa, 56126 Pisa, Italy; elisabetta.fini7@gmail.com

[5] Unit of Medical Statistics, Department of Clinical and Experimental Medicine, University of Pisa, 56126 Pisa, Italy; sabrina.giometto@gmail.com (S.G.); ersilia.lucenteforte@unipi.it (E.L.)

[6] Unit of Adverse Drug Reactions Monitoring, University Hospital of Pisa, 56126 Pisa, Italy

* Correspondence: m.tuccori@ao-pisa.toscana.it or marco.tuccori@gmail.com; Tel.: +39-050-2218761; Fax: +39-050-992071

Supplementary Material (SM) Figures and Tables

Figure S1. First cohort

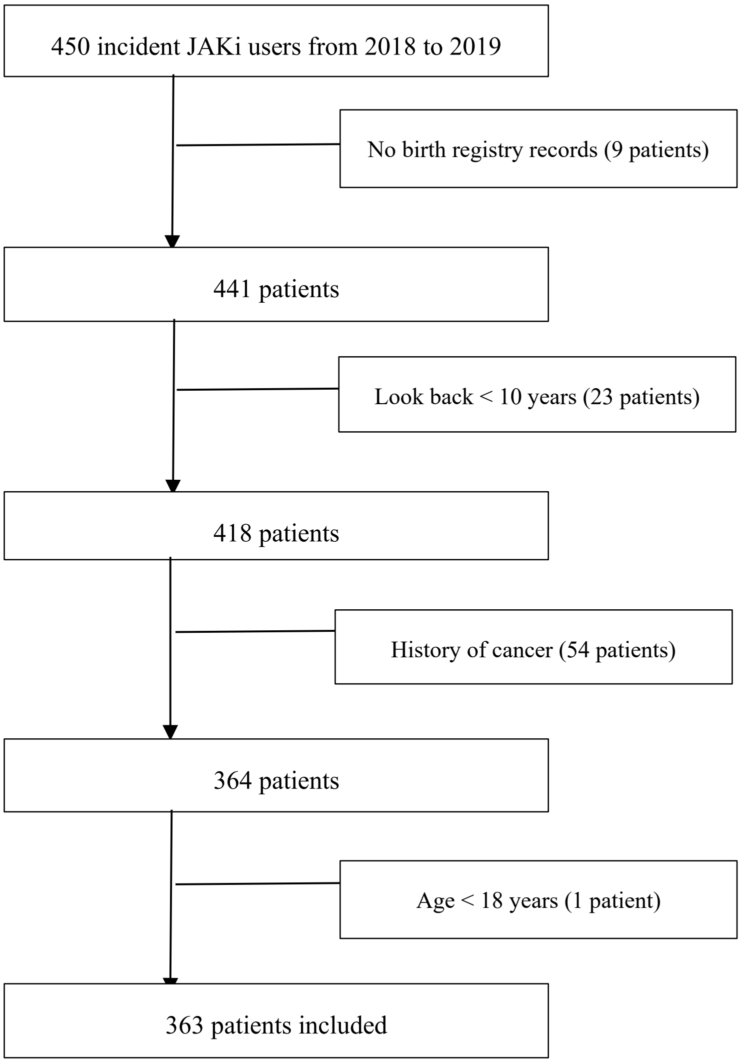

Table S1. DMARDs use history in incident JAKi users

DMARDs	Patients, n (%) (n = 363)
No use	29 (8.0)
Any use	334 (92.0)
**csDMARDs**	287 (79.1)
Methotrexate (L01BA01)	152 (41.9)
Leflunomide (L04AA13)	121 (33.3)
Azathioprine (L04AX01)	10 (2.8)
Cyclosporin (L04AD01)	15 (4.1)
Hydroxychloroquine (P01BA02)	161 (44.4)
Minocycline (A01AB23)	0 (0.0)
Mycophenolate (L04AA06)	2 (0.6)
Sulfasalazine (A07EC01)	39 (10.7)
**anti-TNF bDMARDs**	217 (59.8)
Adalimumab (L04AB04)	97 (26.7)
Certolizumabpegol (L04AB05)	60 (16.5)
Etanercept (L04AB01)	147 (40.5)
Golimumab (L04AB06)	42 (11.6)
Infliximab (L04AB02)	28 (7.7)
**MOA bDMARDs**	152 (41.9)
Abatacept (L04AA24)	110 (30.3)
Rituximab (L01XC02)	34 (9.4)
Tocilizumab (L04AC07)	74 (20.4)
Sarilumab (L04AC14)	0 (0.0)

bDMARDs: biologic DMARDs; csDMARDs: conventional synthetic DMARDs; DMARDs: disease modifying anti-rheumatic drugs; JAKi: Janus Kinase inhibitor; MOA: alternative mechanism of action from TNF inhibitor; TNF: tumor necrosis factor

Table S2. History of DMARDs dispensations

DMARD	Overall dispensation, n	Dispensation per patients, mean (SD)	Dispensations groups, n (%)		
			1-3	4-9	≥10
Conventional sysnteticDMARDs (csDMARDs) (n =500)*					
Methotrexate (n = 152)	3451	22.7 (23.6)	33(22)	26 (17)	93 (61)
Leflunomide (n = 121)	2261	18.7 (22.0)	33 (27)	32 (27)	56 (46)
Azathioprine (n = 10)	172	17.2 (16.5)	3 (30)	1 (10)	6 (60)
Cyclosporin (n =15)	102	6.8 (9.9)	7 (47)	6 (40)	2 (13)
Hydroxychloroquine (n =161)	2398	14.9 (17.8)	54 (33)	30 (19)	77 (48)
Minocycline (n = 0)	0	0 (0.0)	0	0	0
Mycophenolate (n = 2)	14	7.0 (7.1)	1 (50)	0 (0)	1 (50)
Sulfasalazine (n = 39)	252	6.5 (8.7)	23 (59)	6 (15)	10 (26)
Anti-TNF biologic DMARDs (n =374)					
Adalimumab (n = 97)	1589	16.4 (20.1)	18 (19)	31 (32)	48 (49)
Certolizumabpegol (n = 60)	893	14.9 (17.7)	16 (27)	21 (35)	23 (38)
Etanercept (n = 147)	3762	25.6 (30.0)	26 (18)	36 (24)	85 (58)
Golimumab (n = 42)	512	12.2 (13.6)	8 (19)	18 (43)	16 (38)
Infliximab (n =28)	306	10.9 (14.9)	11 (39)	10 (36)	7 (25)
MOA biologic DMARDs (n =218)					
Abatacept (n = 110)	1843	16.8 (18.2)	29 (26)	28 (26)	53 (48)
Rituximab (n = 34)	234	6.9 (6.4)	14 (41)	11 (32)	9 (27)
Tocilizumab (n =74)	1734	23.4 (30.7)	7 (9)	26 (35)	41 (56)
Sarilumab (n = 0)	0	0 (0.0)	0	0	0

bDMARDs: biologic DMARDs; csDMARDs: conventional synthetic DMARDs; DMARDs: disease modifying anti-rheumatic drugs; JAKi: Janus Kinase inhibitor; MOA: alternative mechanism of action from TNF inhibitor; n: number; SD: standard deviation; TNF: tumor necrosis factor

* number of patients with at least a dispensation

Table S3. Distribution of DMARD and bDMARD users among patients receiving the first JAKi stratified for time periods

Time periods	Patients with first DMARD, n, (%) n=334	Patients with first bDMARD, n (%) n =253
< 1 year	20 (6)	47 (19)
1 ≤ years < 2	20 (6)	26 (10)
2 ≤ years < 3	24 (7)	29 (11)
3 ≤ years < 5	26 (8)	41 (16)
5 ≤ years < 10	244 (73)	110 (44)

DMARDs: disease modifying anti-rheumatic drugs; bDMARD: biologic DMARD; DMARDs: disease modifying anti-rheumatic drugs; JAKi: Janus Kinase inhibitor; n: number

Figure S2. Second cohort

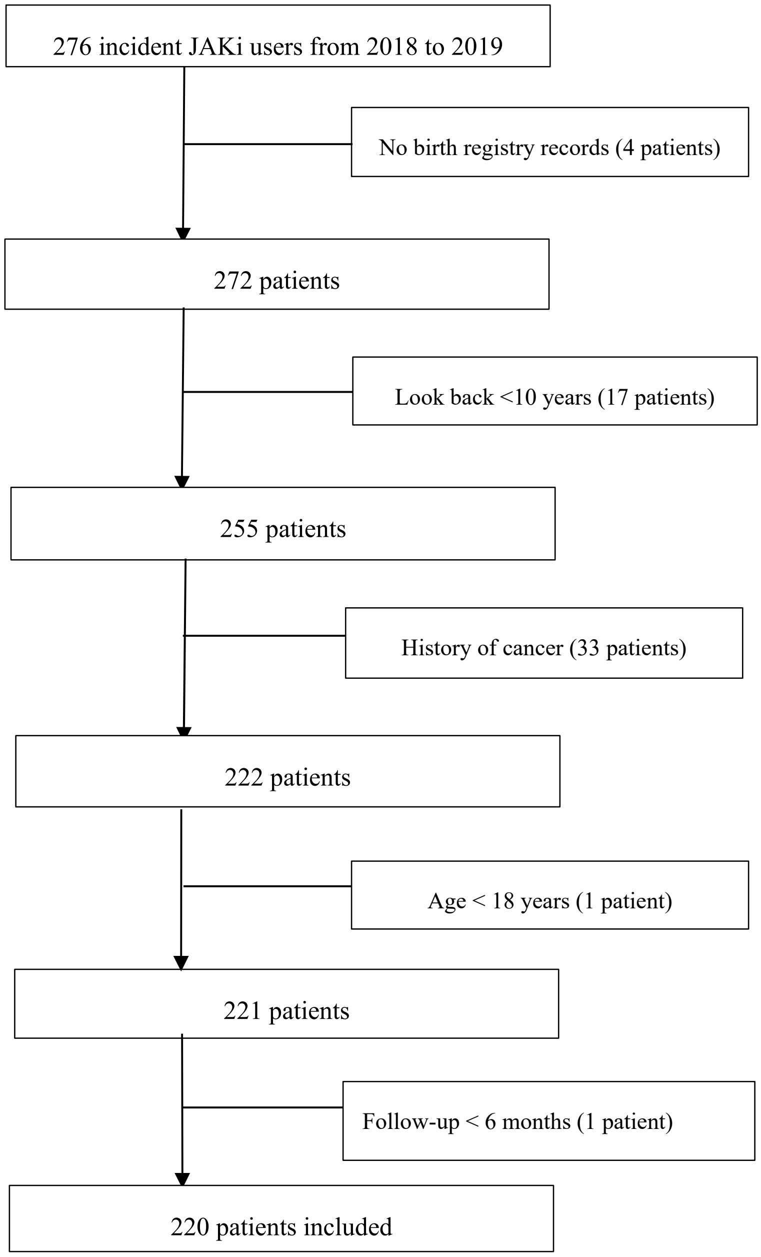

	Overall (n)	Baricitinib (n)	Tofacitinib (n)
Users	220	178	42
ED admissions (n)	109	68	41
Hospitalizations (n)	39	28	11
RA visits (n)	64	64	0

ED: emergency department; n: number; RA: rheumatoid arthritis

Table S4. Distribution of healthcare services assessed in the follow-up

Table S5. Diagnoses associated with the ED admissions in the follow up

Description	ICD-9 code	Cases, n (%)
Injury and poisoning	800-999	20 (18.3)
Diseases of the skin and subcutaneous tissue	680-709	15 (13.8)
Diseases of the circulatory system	390-459	12 (11.0)
Symptoms, signs, and ill-defined conditions	780-799	10 (9.2)
Diseases of the nervous system and sense organs	320-389	10 (9.2)
Diseases of the digestive system	520-579	8 (7.3)
Diseases of the musculoskeletal system and connective tissue	710-739	7 (6.4)
Diseases of the respiratory system	460-519	5 (4.6)
Diseases of the genitourinary system	580-629	3 (2.8)
Supplementary classification of factors influencing health status and contact with health services	V01-V91	2 (1.8)
Infectious and parasitic diseases	001-139	2 (1.8)
Mental disorders	290-319	1 (0.9)
Complications of pregnancy, childbirth, and the puerperium	630-679	1 (0.9)
Diseases of the blood and blood-forming organs	280-289	1 (0.9)
Endocrine, nutritional and metabolic diseases, and immunity disorders	240-279	1 (0.9)

JAKi: Janus Kinase inhibitor ED: emergency department

Table S6. Diagnoses associated with hospitalizations in the follow up

Description	ICD-9 code	Cases, n (%)
Diseases of the circulatory system	390-459	27 (69.2)
Diseases of the musculoskeletal system and connective tissue	710-739	25 (64.1)
Diseases of the skin and subcutaneous tissue	680-709	15 (38.5)
Diseases of the respiratory system	460-519	13 (33.3)
Diseases of the nervous system and sense organs	320-389	10 (25.6)
Endocrine, nutritional and metabolic diseases, and immunity disorders	240-279	8 (20.5)
Supplementary classification of factors influencing health status and contact with health services	V01-V91	6 (15.4)
Diseases of the genitourinary system	580-629	5 (12.8)
Infectious and parasitic diseases	001-139	5 (12.8)
Diseases of the blood and blood-forming organs	280-289	5 (12.8)
Diseases of the digestive system	520-579	2 (5.1)
Mental disorders	290-319	2 (5.1)
Neoplasms	140-239	2 (5.1)

JAKi: Janus Kinase inhibitor

Table S7. Direct healthcare costs for the JAKi use in the follow up

	Cost (€)
**Total cost***	**1,054,530**
Drugs	887,946
Hospitalization	165,624
RA visits	960
**Mean (Min;Max) cost per patient**	**4,793.3 (607.5;50,306)**
Drugs	4,036.1 (607.5;8,387.9)
Hospitalization	752.8 (0;43,811)
RA visits	4.4 (0;60)

* Costs of ED accesses are not included; RA: rheumatoid arthritis

Table S8. Study drugs

Drug class	Drug name	ATC code
Targeted synthetic DMARDs (tsDMARDs) – JAKi	Tofacitinib	L04AA29
	Baricitinib	L04AA37
Conventional synthetic DMARDs (csDMARDs)	Methotrexate	L01BA01
	Leflunomide	L04AA13
	Azathioprine	L04AX01
	Cyclosporin	L04AD01
	Hydroxychloroquine	P01BA02
	Minocycline	A01AB23
	Mycophenolate	L04AA06
	Sulfasalazine	A07EC01
Anti-TNF biologic DMARDs (bDMARDs)	Adalimumab	L04AB04
	Certolizumabpegol	L04AB05
	Etanercept	L04AB01
	Golimumab	L04AB06
	Infliximab	L04AB02
MOA biologic DMARDs (bDMARDs)	Abatacept	L04AA24
	Rituximab	L01XC02
	Tocilizumab	L04AC07
	Sarilumab	L04AC14

ATC: anatomical therapeutic chemical classification; DMARDs: disease modifying anti-rheumatic drugs; JAKi: Janus Kinase inhibitor; MOA: alternative mechanism of action from TNF inhibitor; TNF: tumor necrosis factor
